# Cisplatin-induced posterior reversible encephalopathy syndrome and successful re-treatment in a patient with non-seminomatous germ cell tumor: a case report

**DOI:** 10.1186/1752-1947-6-409

**Published:** 2012-11-29

**Authors:** Muhammad Nauman Zahir, Nehal Masood, Munira Shabbir-Moosajee

**Affiliations:** 1 Department of Medicine, Division of Hematology and Oncology, Aga Khan University Hospital, Stadium Road, PO BOX: 3500, Karachi 74800, Pakistan

**Keywords:** Cisplatin, PRES, Re-treatment

## Abstract

**Introduction:**

Cisplatin is a platinum compound that has revolutionized the treatment of various solid organ tumors. Cisplatin is associated with a variety of side effects and has recently been indicted in the development of posterior reversible encephalopathy syndrome. Posterior reversible encephalopathy syndrome is a potentially reversible condition, with the mainstay of therapy being correction of the underlying cause and withdrawal of the offending drug. However, there are no clear guidelines regarding the possibility of subsequent re-treatment with the causative agent.

**Case presentation:**

A 23-year-old Asian man presented to our Emergency Department with a four-month history of concomitant abdominal pain and backache and a two-week history of left-sided leg swelling. Diagnostic investigations revealed bilateral pulmonary embolism, extensive deep venous thrombosis and widespread lung and liver metastatic deposits with abdomino-pelvic lymphadenopathy. His biopsy and tumor markers were consistent with non-seminomatous germ cell tumor and he was subsequently started on an initial cycle of cisplatin and etoposide chemotherapy. On the second day of treatment he developed posterior reversible encephalopathy syndrome clinically and radiologically. Cisplatin was stopped for the next two days while etoposide was continued, resulting in complete resolution of his symptoms. He was re-challenged with cisplatin on day five of chemotherapy because a platin-based chemotherapy regimen was his only hope of potential cure. He tolerated it well, with no recurrence of his neurological symptoms and the remainder of his in-patient stay remained uneventful. He was discharged on day eight. He has since then completed treatment and is currently in remission.

**Conclusions:**

The occurrence of posterior reversible encephalopathy syndrome after cisplatin use has been well reported in the literature. We strongly believe that our patient also developed posterior reversible encephalopathy syndrome secondary to cisplatin. The uniqueness of our patient’s case lies in the successful re-treatment of our patient with the offending drug. To the best of our knowledge, this is the first instance where a patient was successfully re-treated with cisplatin after having developed posterior reversible encephalopathy syndrome as a result of cisplatin use. The excellent response to re-treatment without recurrence of neurological symptoms in our patient’s case provides insight into re-treatment as an option in scenarios where treatment options are limited.

## Introduction

Cisplatin is a platinum compound which has revolutionized the treatment of various solid organ tumors and since its introduction in the 1970s has become the backbone of many chemotherapeutic regimens. Cisplatin is associated with a variety of side effects, the most common being nausea, vomiting, nephrotoxicity, ototoxicity and neurotoxicity [1]. The neurotoxicity usually manifests in the form of an axonal sensory neuropathy as peripheral nerves are exposed to the highest levels of cisplatin with resultant deposition of metal metabolites in these nerves [2]. Central nervous system (CNS) neurotoxicity of cisplatin is rare, but has been well documented in the medical literature. An array of CNS disorders including seizures, hemiparesis, cortical blindness, aphasia and coma have been attributed to cisplatin therapy [3]. Recently, cisplatin has been reported to cause posterior reversible encephalopathy syndrome (PRES).

PRES is a clinico-radiological entity characterized by the onset of a variety of neurological manifestations associated with transient changes typically in the posterior circulation of the brain [2]. The commonly associated neurological symptoms include headache, seizures, confusion and visual disturbances. The exact pathogenesis of PRES remains unclear; however, two factors are known to predispose patients to the development of PRES: disordered cerebral autoregulation and endothelial damage. PRES secondary to cytotoxic chemotherapy is thought to be precipitated by the direct toxic effect of the drug on the cerebral vascular endothelium. Endothelial damage results in capillary leakage, disruption of the blood brain barrier, axonal swelling and development of vasogenic edema, which is a hallmark feature of PRES. Vasogenic edema and autoregulatory failure of the cerebral circulation in turn can lead to a decrease in cerebral perfusion which results in the clinical manifestations of PRES [4,5]. Clinically, it is characterized by subacute development of headache, seizures, confusion and visual disturbance and occasionally hypertensive crisis [2]. These symptoms occur as a result of vasogenic edema in the parietal and occipital lobes, which are preferentially involved [4].

Radiographically, PRES presents on magnetic resonance imaging (MRI) scans as edema symmetrically affecting the subcortical white matter most commonly in the posterior cerebral hemispheres. It is important to note, however, that any region of the CNS can be affected [5,6]. The most common MRI abnormality is punctate or confluent areas of increased signal on proton density and T2-weighted and fluid attenuated inversion recovery (FLAIR) images. Additional MRI features can include gyriform signal enhancement following the administration of gadolinium, petechial and large parenchymal hemorrhages, hypo or iso-intense signal on diffusion weighted imaging and increased signal on apparent diffusion coefficient (ADC) maps [7].

A wide variety of medical conditions have been implicated with PRES. The more frequently reported association is with hypertensive encephalopathy, eclampsia and use of immunosuppressive drugs [5]. Various cytotoxic, immunosuppressive and biological agents including cisplatin have been indicted in the development of PRES [8]. PRES, as the name suggests, is a potentially reversible condition, with the fundamental step in treatment being correction of the underlying cause and withdrawal of the offending drug. However, there are no clear guidelines on the possibility of re-treatment with the causative agent.

## Case presentation

We report the case of a 23-year-old Asian man who presented to our Emergency Department (ED) with a four-month history of concomitant abdominal pain and backache and a two-week history of left-sided leg swelling. An abdominal ultrasonogram performed for his complaints revealed a soft tissue mass involving the prostate, abutting the anterior rectal wall and the urinary bladder along with significant para-aortic lymphadenopathy.

He was in respiratory distress on presentation to the ED. Further investigation showed bilateral pulmonary embolism due to extensive deep venous thrombosis (DVT) involving both lower limbs and extending up to the inferior vena cava (IVC) for which low-molecular-weight heparin (LMWH) was initiated. Computed tomography (CT) scans additionally revealed extensive lung and liver metastatic deposits along with conglomerate abdomino-pelvic lymphadenopathy. An ultrasonogram of the testes was negative for any intra-testicular or extra-testicular mass lesion. Given his relatively young age at presentation, a retro-peritoneal germ cell tumor was suspected.

The serum lactate dehydrogenase (LDH), α-fetoprotein (AFP) and β-human chorionic gonadotropin (β-HCG) levels were 7596IU/L, 88.5IU/mL and 21,753mIU/mL, respectively. Biopsy of the abdominal adenopathy was consistent with a non-seminomatous germ cell tumor with a prominent yolk sac tumor component. Pulmonary function tests with diffusing capacity of the lung for carbon monoxide (DLCO) were performed, which revealed the DLCO to be only 46 percent of the predicted value.

**Figure 1:**
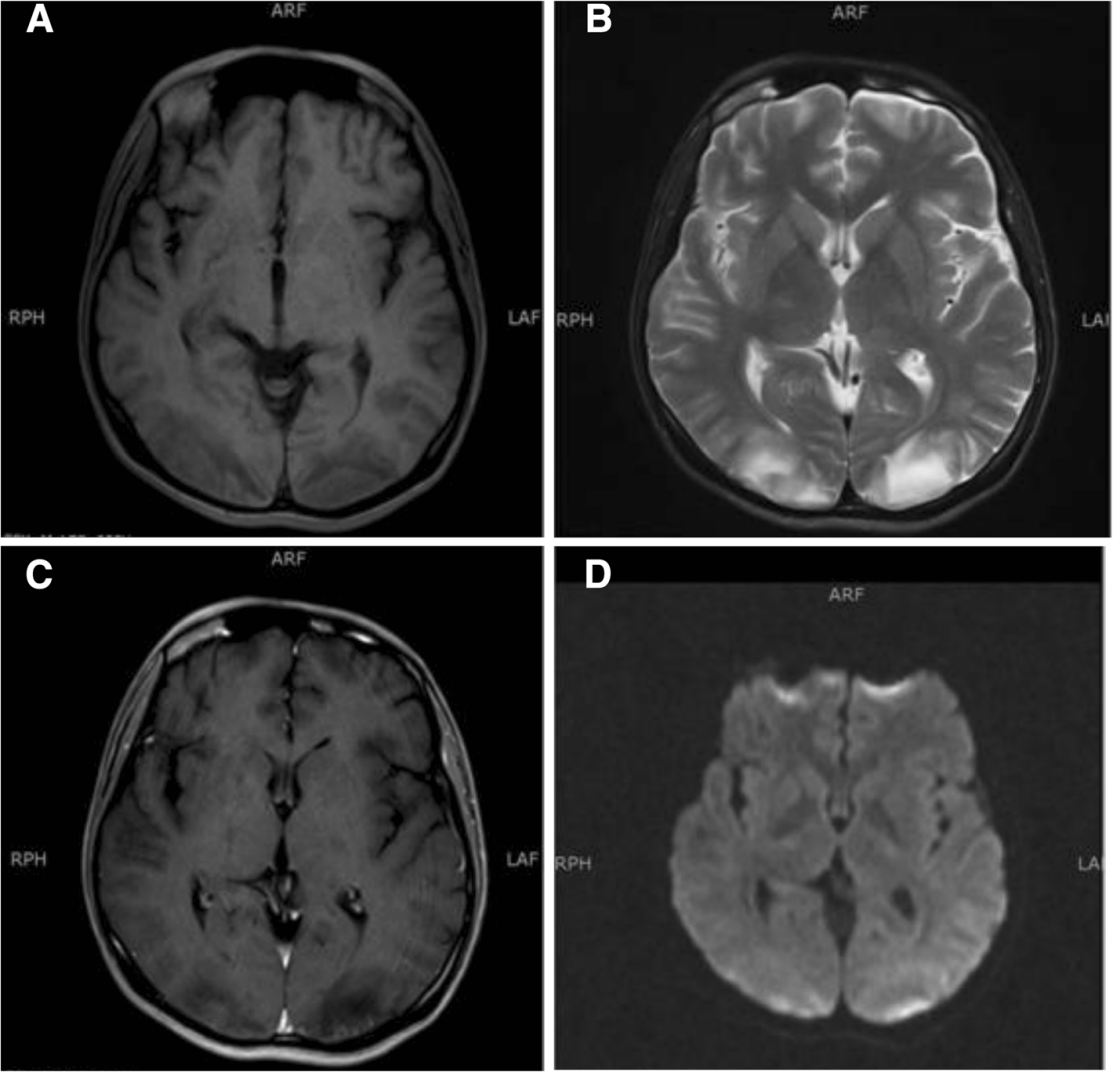
**Posterior reversible encephalopathy syndrome after cisplatin treatment.** Bilateral and symmetrical abnormal intensity areas are identified in the occipital lobes involving the cortical and subcortical locations. These are hypointense on T1-weighted images **(A)** and hyperintense on T2-weighted images **(B)**, showing minimal patchy post-contrast enhancement **(C)** with no diffusion restriction **(D)**.

Our patient was established as having a stage IIIC (poor risk) non-seminomatous germ cell tumor and was subsequently started on cisplatin (20mg/m^2^ on days one to five) and etoposide (100mg/m^2^ on days one to five). Bleomycin was omitted from the regimen due to the poor DLCO. On the evening of day two of chemotherapy, he started having hypertensive episodes with systolic blood pressures ranging from 150 to 170mmHg and diastolic pressures in the range of 100 to 110mmHg. Within a few hours, he developed a tonic clonic seizure that was aborted by intravenous diazepam. He was also started on calcium channel blockers for management of hypertension. He then started to complain of persistent blurring of vision once fully conscious from the post-ictal phase. His pupillary reflexes and fundoscopy were normal. A neurological examination revealed hyper-reflexia of the lower limbs. Investigations excluded a metabolic abnormality as the cause of seizure. An MRI scan of the brain showed multiple abnormal signal intensity areas involving the peri-ventricular, posterior parietal and occipital regions bilaterally which were hyperintense on T2-weighted images, hypointense on T1-weighted images and showed patchy post-contrast enhancement with no diffusion restriction (Figure 1). The findings were reported to be consistent with PRES. Cisplatin was withheld for the next two days as it was thought to be the most probable cause of PRES, and chemotherapy with single agent etoposide was continued. At 48 hours after the seizure and the withdrawal of cisplatin, our patient was significantly better with no further seizure episodes and almost complete resolution of his visual symptoms. His blood pressure had also been controlled with anti-hypertensive medications.

At this point we decided to resume cisplatin, because a platinum-based regimen was his only hope of potential cure. Under close surveillance, our patient was re-challenged with cisplatin at 20mg/m^2^ on day five of chemotherapy. He tolerated it well with no recurrence of neurological symptoms, and the remainder of his in-patient stay remained uneventful. He was discharged on day eight of chemotherapy with complete resolution of symptoms.

**Figure 2:**
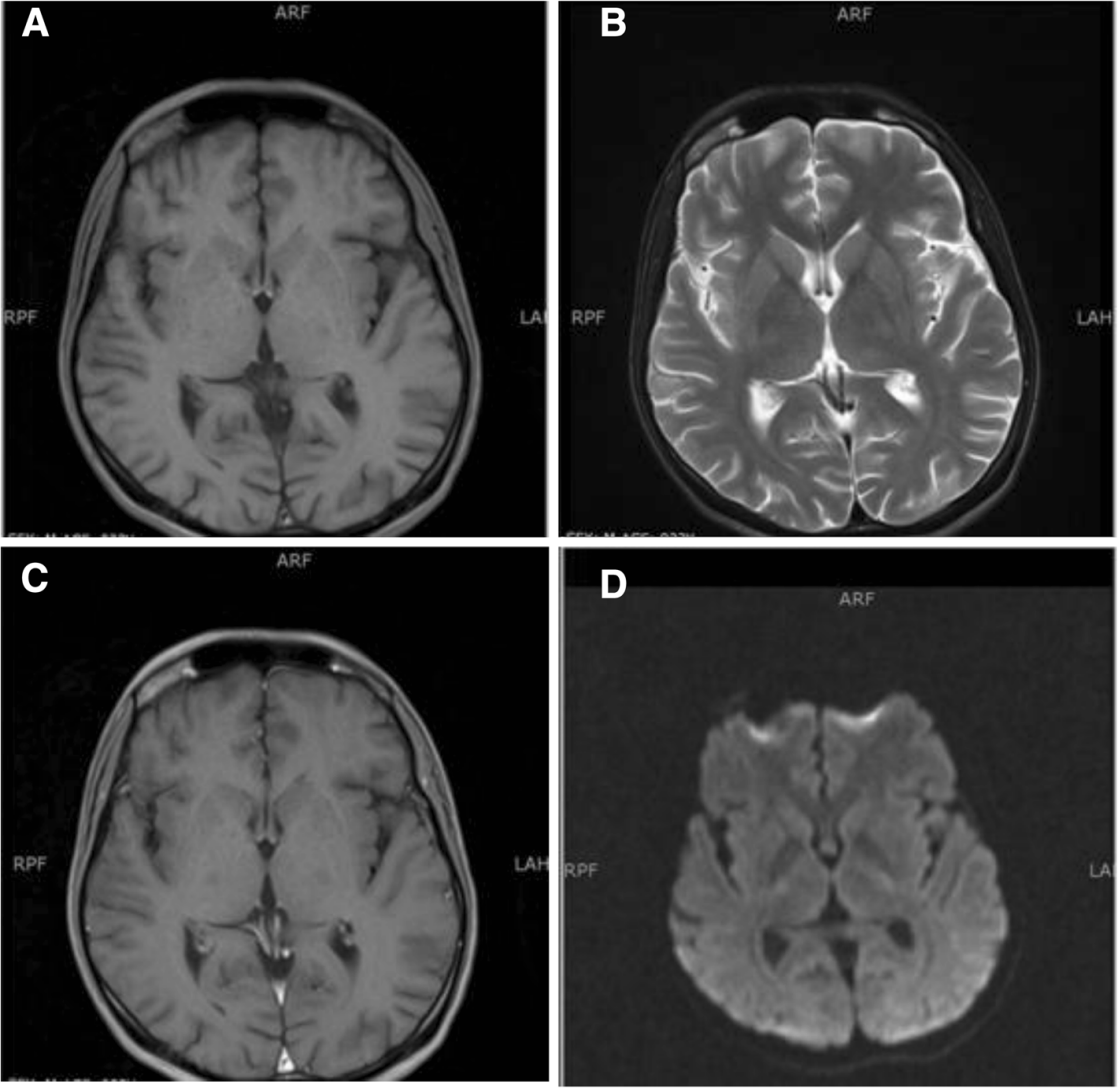
**Magnetic resonance imaging scan of the brain six weeks after initial treatment with cisplatin.** The changes of posterior reversible encephalopathy syndrome have resolved in both T1-weighted and T2-weighted images **(A,B)**. The T1 post-contrast **(C)** and diffusion-weighted images **(D)** are also normal.

He then went on to complete three additional cycles of cisplatin and etoposide with no dose reductions, with no further neurological complications. An interim analysis of disease response after two cycles was significant for normalization of LDH and β-HCG levels and a greater than 95 percent decline in AFP. CT scans showed significant improvement in disease process. Results of a brain MRI scan performed at the same time as this follow up were normal (Figure 2).

Post-treatment CT scans showed complete resolution of his liver and lung lesions but a residual retro-peritoneal nodal mass. Tumor markers (LDH, AFP and β-HCG) had all returned to within normal limits. As per international guidelines, he subsequently underwent resection of the residual nodal mass. Pathology was consistent with fibrocollagenous tissue exhibiting extensive fibrosis, but no viable tumor.

He is currently 90 days post-surgery and has recovered well. Post-operative scans show no evidence of disease. His tumor markers continue to remain within normal limits.

## Conclusions

In the cases of PRES due to cisplatin reported to date, development of the syndrome led to discontinuation of cisplatin with resultant resolution of the symptoms. We strongly believe that our patient also developed PRES secondary to cisplatin. The fact that his symptoms resolved completely after withdrawal of cisplatin despite continuation of etoposide, which was the only other chemotherapeutic drug in the regimen, led us to this conclusion.

The uniqueness of our case does not lie in the development of PRES secondary to cisplatin use, but rather in the successful re-treatment of our patient with the offending drug once the acute symptomatology had subsided. The decision to re-treat with cisplatin was taken because platinum based regimens form the backbone of treatment of germ cell tumors. It was decided that in our patient’s case permanent withdrawal of cisplatin from the regimen would severely compromise our efforts of effectively treating a potentially curable malignancy. Carboplatin could have been used as an alternative to cisplatin for further treatment, but has been shown to be inferior in efficacy to the former [9] and was hence not contemplated.

To the best of our knowledge, this is the first instance when a patient was successfully re-treated with cisplatin after having developed PRES. The only other example in the literature is of re-treatment with bevacizumab in a patient with glioblastoma [10].

The excellent response to re-treatment without recurrence of neurological symptoms in our patient’s case provides insight that the possibility of re-treatment with the offending agent may be an option in scenarios where other interventions are limited. In the field of medical oncology where curative therapeutic options may sometimes be very limited, this possibility of re-treatment could be invaluable. However, the ultimate safety of this approach will require additional evaluation.

## Consent

Written informed consent was obtained from the patient for publication of this case report and any accompanying images. A copy of the written consent is available for review by the Editor-in-Chief of this journal.

## Competing interests

The authors declare that they have no competing interests.

## Authors’ contributions

MNZ performed the literature search and drafted the manuscript. NM conceived the case report and provided guidance for drafting the manuscript. MSM participated in its design and co-ordination and helped to draft the manuscript. All authors read and approved the final manuscript.
